# 2-Propoxybenzamide

**DOI:** 10.1107/S1600536812033326

**Published:** 2012-08-04

**Authors:** Yosef Al Jasem, Bassam al Hindawi, Thies Thiemann, Fraser White

**Affiliations:** a Department of Chemical Engineering, United Arab Emirates University, AL Ain, Abu Dhabi, United Arab Emirates; b Department of Chemistry, United Arab Emirates University, AL Ain, Abu Dhabi, United Arab Emirates; c Agilent Technologies UK Ltd, 10 Mead Road, Oxford Industrial Park, Oxford OX5 1QU, England

## Abstract

In the title mol­ecule, C_10_H_13_NO_2_, the amide –NH_2_ group is oriented toward the prop­oxy substituent and an intra­molecular N—H⋯O hydrogen bond is formed between the N—H group and the prop­oxy O atom. The benzene ring forms dihedral angles of 12.41 (2) and 3.26 (2)° with the amide and prop­oxy group mean planes, respectively. In the crystal, N—H⋯O hydrogen bonds order pairs of mol­ecules with their mol­ecular planes parallel, but at an offset of 0.73 (2) Å to each other. These pairs are ordered into two types of symmetry-related columns extended along the *a* axis with the mean plane of a pair in one column approximately parallel to (-122) and in the other to (-1-22). The two planes form dihedral angle of 84.40 (1)°. Overall, in a three-dimensional network, the hydrogen-bonded pairs of mol­ecules are either located in (-1-22) or (-122) layers. In one layer, each pair is involved in four C—H⋯O contacts, twice as a donor and twice as an acceptor. Additionally, there is a short C—H⋯C contact between a benzene C—H group and the amide π-system.

## Related literature

For crystal structures of similar compounds, see: Pagola & Stephens (2009 ▶); Johnstone *et al.* (2010 ▶); Pertlik *et al.* (1990 ▶); Sasada *et al.* (1964 ▶). For uses of 2-alk­oxy­benzamides, see: van de Waterbeemd & Testa (1983 ▶); Kusunoki & Harada (1984 ▶). For the preparation of the title compound, see: Johnstone & Rose (1979 ▶).
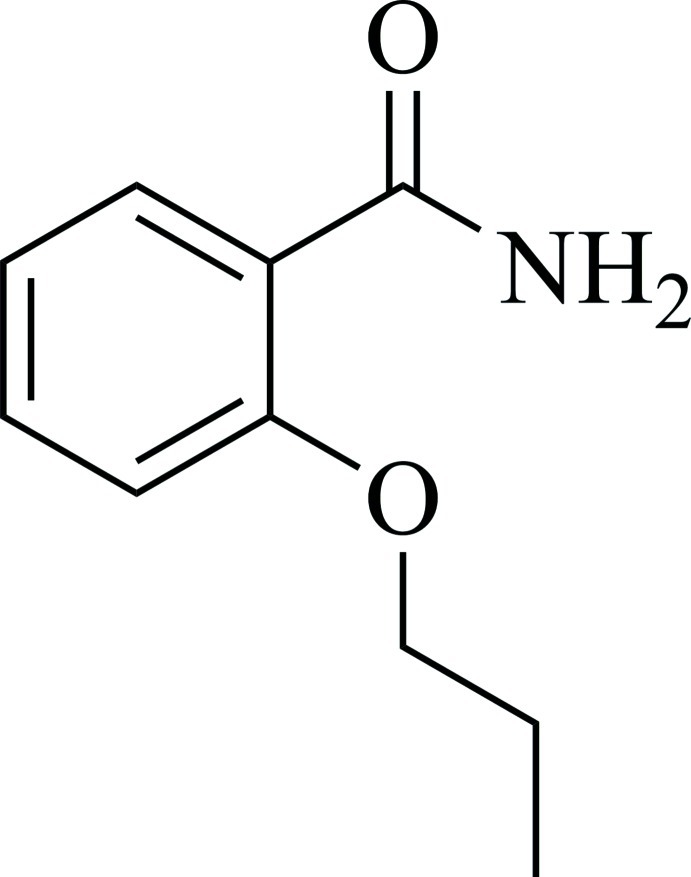


## Experimental

### 

#### Crystal data

C_10_H_13_NO_2_
*M* *_r_* = 179.21
Monoclinic, 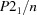

*a* = 6.0303 (4) Å
*b* = 11.1196 (8) Å
*c* = 14.4140 (11) Å
β = 98.647 (6)°
*V* = 955.54 (12) Å^3^
*Z* = 4
Cu *K*α radiation
μ = 0.71 mm^−1^
*T* = 100 K
0.16 × 0.10 × 0.08 mm

#### Data collection

Agilent SuperNova Atlas CCD diffractometer
Absorption correction: gaussian (*CrysAlis PRO*; Agilent, 2012 ▶) *T*_min_ = 0.810, *T*_max_ = 1.000
2959 measured reflections
1676 independent reflections
1253 reflections with *I* > 2σ(*I*)
*R*_int_ = 0.035

#### Refinement

*R*[*F* ^2^ > 2σ(*F* ^2^)] = 0.040
*wR*(*F* ^2^) = 0.099
*S* = 1.03
1676 reflections
171 parameters
All H-atom parameters refined
Δρ_max_ = 0.21 e Å^−3^
Δρ_min_ = −0.17 e Å^−3^

### 

Data collection: *CrysAlis PRO* (Agilent, 2012 ▶); cell refinement: *CrysAlis PRO*; data reduction: *CrysAlis PRO*; program(s) used to solve structure: *SHELXS97* (Sheldrick, 2008 ▶); program(s) used to refine structure: *SHELXL97* (Sheldrick, 2008 ▶) within *OLEX2* (Dolomanov *et al.*, 2009 ▶); molecular graphics: *PLATON* (Spek, 2009 ▶) and *Mercury* (Macrae *et al.*, 2008 ▶); software used to prepare material for publication: *SHELXL97* and *PLATON* (Spek, 2009 ▶).

## Supplementary Material

Crystal structure: contains datablock(s) global, I. DOI: 10.1107/S1600536812033326/gk2512sup1.cif

Structure factors: contains datablock(s) I. DOI: 10.1107/S1600536812033326/gk2512Isup2.hkl

Supplementary material file. DOI: 10.1107/S1600536812033326/gk2512Isup3.cml

Additional supplementary materials:  crystallographic information; 3D view; checkCIF report

## Figures and Tables

**Table 1:** Hydrogen-bond geometry (Å, °)

*D*—H⋯*A*	*D*—H	H⋯*A*	*D*⋯*A*	*D*—H⋯*A*
N13—H13*A*⋯O7	0.94 (2)	1.95 (2)	2.669 (2)	132.2 (15)
N13—H13*B*⋯O12^i^	0.93 (2)	1.98 (2)	2.911 (2)	173.5 (19)
C6—H6⋯O12^ii^	0.927 (19)	2.517 (18)	3.442 (2)	175.8 (14)
C5—H5⋯C11^iii^	0.94 (2)	2.63 (2)	3.528 (2)	160.5 (17)

Symmetry codes: (i) 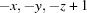; (ii) 
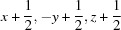
; (iii) 
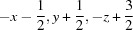
.

**Fig. 1.**
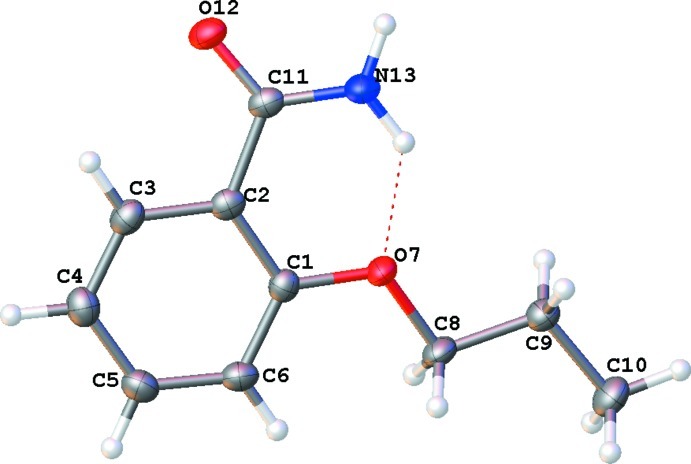
A view of the title compound molecule with the atom-numbering scheme and the intramolecular interaction within the molecule. Displacement ellipsoids are shown at the 50% probability level.

**Fig. 2.**
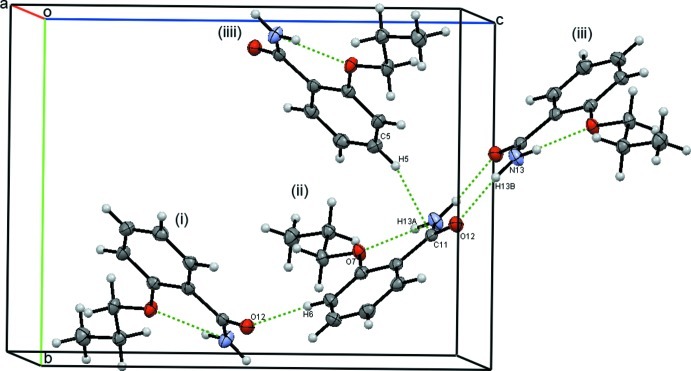
Intermolecular and intramolecular attractions between molecules of the title compound. [Symmetry codes: (i): -x,1 - y,1 - z (ii): 1/2 - x,1/2 + y,1.5 - z (iii): 1/2 + x,1/2 - y,1/2 + z (iiii): 1 + x,y,z]

**Fig. 3.**
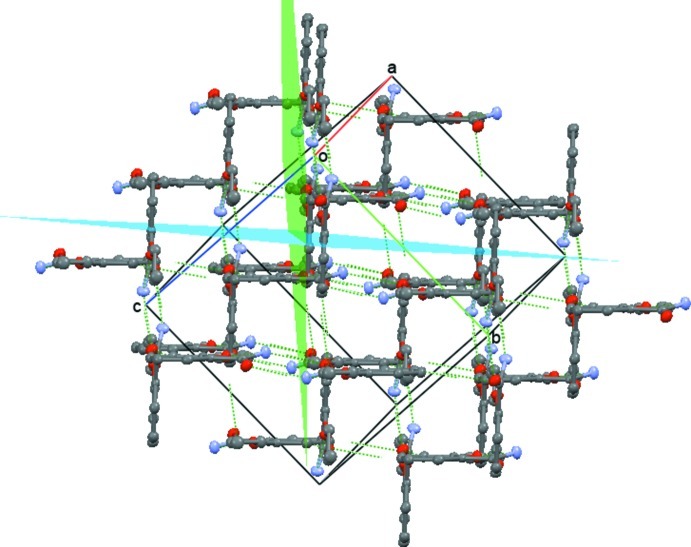
The crystal network formed by the layers of molecular pairs in the planes (-1 2 2) in blue, and (-1 –2 2) in green.

## supplementary crystallographic information

### Comment

In the 2-propoxybenzamide molecule, the C3—C2—C11—O12 torsion angle characterizing the twist of the benzene ring relative to the amide group is -11.5 (2)° and corresponding C6—C1—O7—C8 angle for the propoxy group is 3.0 (2)°. There is an intramolecular N13—H13A···O7 hydrogen bond within each molecule. The hydrogen bond N13—H13B···O12 (Table 1) plays a significant role in the packing of the title compound, forming pairs of inversion related molecules, with the molecular planes in parallel (Figure 2). These pairs form a nested network crystal, made of two layers, (-1 -2 2) and (-1 2 2), which form an angle of 84.40 (1)° between their planes (Figure 3). The molecules in layers are linked to each other by C6—H6···O12 interaction (Table 1). Within two parallel layers, pairs are lying with an offset to each other without any noticeable, direct interaction between them. The parallel layers are at a distance of 3.69 (2) Å from each other. They are further apart than is found for a similar packing of 2-hydroxybenzamide [2.91 (1) Å] (Johnstone *et al.*, 2010). Along the *a* axis the pairs are ordered in two symmetry related columns. The plane of the benzene ring (C1—C6) of the 2-propoxybenzamide forms an angle of 34.43 (2)° with the column axis.

For 2-ethoxybenzamide, a homologue of the title compound, a similar formation of inversion related molecular pairs in the crystal was reported (Pagola & Stephens, 2009). The noticable difference in the packing of the two molecules stems from the larger dihedral angle between the carboxamide group and the benzene ring in 2-ethoxybenzamide of [50.48 (2)°] than found for 2-propoxybenzamide. Thus, 2-ethoxybenzamide does not exhibit an intramolecular hydrogen bond, which leads to a different intermolecular bonding network as compared to the title compound.

### Experimental

To powdered KOH (1.12 g, 20.0 mmol) in DMSO (9 ml) was added salicylamide (1.37 g, 10.0 mmol), and the resulting mixture was stirred for 10 min. at rt. Thereafter, *n*-propyl iodide (4.2 g, mmol, 24.7 mmol) was added dropwise. The solution was stirred for 12 h at rt. Then, it was poured into water (200 ml) and extracted with chloroform (3 x 50 ml). The organic phase was dried over anhydrous MgSO_4_, concentrated *in vacuo*, and the residue was subjected to column chromatography on silica gel (CHCl_3_/*M^t^*BE 1:1) to give 2-propoxybenzamide (1.36 g, 77%) as colorless crystals (m.p. 375 K). The crystal was grown from chloroform/ *M^t^*BE (*v/v* 1:1). IR (KBr) *n*_max_ 3445, 3325, 3273, 3180, 2964, 2935, 2877, 1665, 1594, 1454, 1378, 1277, 1237, 1042, 1010, 759, 566 cm^-1^; ^1^H NMR (DMSO-d^6^, 400 MHz) 0.97 (3*H*, t, ^3^*J* = 7.6 Hz), 1.76 (2*H*, qt, ^3^*J* = 7.6 Hz, ^3^*J* = 6.4 Hz), 4.04 (2*H*, t, ^3^*J* = 6.4 Hz), 6.99 (dd, ^3^*J* = 8.0 Hz, ^3^*J* = 8.0 Hz), 7.10 (1*H*, d, ^3^*J* = 8.0 Hz), 7.43 (1*H*, ddd, ^3^*J* = 8.0 Hz, ^3^*J* = 8.0 Hz, ^4^*J* = 2.0 Hz), 7.78 (1*H*, dd, ^3^*J* = 8.0 Hz, ^4^*J* = 2.0 Hz); ^1^H NMR (CDCl_3_, 400 MHz) 1.07 (3*H*, t, ^3^*J* = 7.4 Hz), 1.90 (2*H*, dd, ^3^*J* = 7.4 Hz, ^3^*J* = 6.5 Hz), 4.09 (2*H*, t, ^3^*J* = 6.5 Hz), 6.07 (1*H*, bs, NH), 6.96 (1*H*, d, ^3^*J* = 8.0 Hz), 7.05 (1*H*, dd, ^3^*J* = 7.8 Hz, ^3^*J* = 7.6 Hz), 7.44 (1*H*, dd, ^3^*J* = 8.0 Hz, ^3^*J* = 7.6 Hz, ^4^*J* = 1.8 Hz), 7.84 (1*H*, bs, NH), 8.20 (1*H*, dd, ^3^*J* = 7.8 Hz, ^4^*J* = 1.8 Hz); ^13^C NMR (DMSO-d^6^, 400 MHz) 11.0, 22.3, 70.4, 113.3, 120.8, 123.1, 131.2, 132.9, 157.0, 166.8; ^13^C NMR (CDCl_3_, 400 MHz) 10.7, 22.6, 71.0, 112.8, 121.4, 121.7, 133.2, 134.0, 158.2, 168.1.

### Refinement

All the H-atom parameters were freely refined.

### Crystal data

**Table:**

C_10_H_13_NO_2_	*D*_x_ = 1.246 Mg m^−^^3^
*M**_r_* = 179.21	Melting point: 375 K
Monoclinic, *P*2_1_/*n*	Cu *K*α radiation, λ = 1.5418 Å
*a* = 6.0303 (4) Å	Cell parameters from 1037 reflections
*b* = 11.1196 (8) Å	θ = 3.1–66.6°
*c* = 14.4140 (11) Å	µ = 0.71 mm^−^^1^
β = 98.647 (6)°	*T* = 100 K
*V* = 955.54 (12) Å^3^	Block, colourless
*Z* = 4	0.16 × 0.10 × 0.08 mm
*F*(000) = 384	

### Data collection

**Table:**

Agilent SuperNova Atlas CCD diffractometer	1676 independent reflections
Radiation source: SuperNova (Cu) X-ray Source	1253 reflections with *I* > 2σ(*I*)
Mirror monochromator	*R*_int_ = 0.035
Detector resolution: 10.4948 pixels mm^-1^	θ_max_ = 66.7°, θ_min_ = 5.1°
ω scans	*h* = −7→7
Absorption correction: gaussian (*CrysAlis PRO*; Agilent, 2012)	*k* = −13→13
*T*_min_ = 0.810, *T*_max_ = 1.000	*l* = −17→15
2959 measured reflections	

### Refinement

**Table:**

Refinement on *F*^2^	Secondary atom site location: difference Fourier map
Least-squares matrix: full	Hydrogen site location: inferred from neighbouring sites
*R*[*F*^2^ > 2σ(*F*^2^)] = 0.040	All H-atom parameters refined
*wR*(*F*^2^) = 0.099	*w* = 1/[σ^2^(*F*_o_^2^) + (0.0471*P*)^2^*P*] where *P* = (*F*_o_^2^ + 2*F*_c_^2^)/3
*S* = 1.03	(Δ/σ)_max_ < 0.001
1676 reflections	Δρ_max_ = 0.21 e Å^−^^3^
171 parameters	Δρ_min_ = −0.17 e Å^−^^3^
0 restraints	Extinction correction: *SHELXL97* (Sheldrick, 2008), Fc^*^=kFc[1+0.001xFc^2^λ^3^/sin(2θ)]^-1/4^
Primary atom site location: structure-invariant direct methods	Extinction coefficient: 0.0017 (4)

### Special details

**Table:**

Geometry. All e.s.d.'s (except the e.s.d. in the dihedral angle between two l.s. planes) are estimated using the full covariance matrix. The cell e.s.d.'s are taken into account individually in the estimation of e.s.d.'s in distances, angles and torsion angles; correlations between e.s.d.'s in cell parameters are only used when they are defined by crystal symmetry. An approximate (isotropic) treatment of cell e.s.d.'s is used for estimating e.s.d.'s involving l.s. planes.
Refinement. Refinement of *F*^2^ against ALL reflections. The weighted *R*-factor *wR* and goodness of fit *S* are based on *F*^2^, conventional *R*-factors *R* are based on *F*, with *F* set to zero for negative *F*^2^. The threshold expression of *F*^2^ > σ(*F*^2^) is used only for calculating *R*-factors(gt) *etc*. and is not relevant to the choice of reflections for refinement. *R*-factors based on *F*^2^ are statistically about twice as large as those based on *F*, and *R*- factors based on ALL data will be even larger.

### Fractional atomic coordinates and isotropic or equivalent isotropic displacement parameters (Å^2^)

**Table:**

	*x*	*y*	*z*	*U*_iso_*/*U*_eq_	
C1	0.0319 (3)	0.24260 (15)	0.75450 (12)	0.0205 (4)	
C10	0.7095 (3)	0.1142 (2)	0.96172 (14)	0.0319 (5)	
C11	−0.0529 (3)	0.13553 (15)	0.59371 (12)	0.0210 (4)	
C2	−0.1026 (3)	0.22312 (15)	0.66721 (12)	0.0206 (4)	
C3	−0.3032 (3)	0.28748 (16)	0.64722 (13)	0.0246 (4)	
C4	−0.3695 (3)	0.36968 (16)	0.70979 (13)	0.0274 (4)	
C5	−0.2326 (3)	0.38823 (16)	0.79474 (13)	0.0263 (4)	
C6	−0.0335 (3)	0.32574 (16)	0.81744 (13)	0.0240 (4)	
C8	0.3557 (3)	0.18715 (17)	0.86630 (12)	0.0240 (4)	
C9	0.5534 (3)	0.10404 (17)	0.86848 (13)	0.0245 (4)	
H10A	0.766 (3)	0.198 (2)	0.9734 (15)	0.049 (7)*	
H10B	0.839 (4)	0.054 (2)	0.9640 (14)	0.047 (6)*	
H10C	0.634 (4)	0.092 (2)	1.0150 (17)	0.051 (7)*	
H13A	0.256 (3)	0.1050 (18)	0.6538 (15)	0.035 (6)*	
H13B	0.175 (3)	0.028 (2)	0.5573 (15)	0.043 (6)*	
H3	−0.394 (3)	0.2739 (16)	0.5883 (13)	0.022 (5)*	
H4	−0.515 (3)	0.4133 (18)	0.6958 (14)	0.040 (6)*	
H5	−0.273 (3)	0.4440 (19)	0.8381 (15)	0.038 (6)*	
H6	0.056 (3)	0.3387 (15)	0.8747 (13)	0.020 (5)*	
H8A	0.408 (3)	0.2707 (16)	0.8776 (13)	0.019 (4)*	
H8B	0.263 (3)	0.1645 (17)	0.9168 (15)	0.034 (5)*	
H9A	0.499 (3)	0.0208 (18)	0.8581 (13)	0.031 (5)*	
H9B	0.635 (3)	0.1262 (16)	0.8153 (14)	0.027 (5)*	
N13	0.1514 (3)	0.08719 (15)	0.60043 (11)	0.0273 (4)	
O12	−0.2027 (2)	0.10985 (11)	0.52757 (8)	0.0268 (3)	
O7	0.22330 (19)	0.17526 (11)	0.77463 (8)	0.0236 (3)	

### Atomic displacement parameters (Å^2^)

**Table:**

	*U*^11^	*U*^22^	*U*^33^	*U*^12^	*U*^13^	*U*^23^
C1	0.0192 (8)	0.0205 (9)	0.0215 (9)	−0.0012 (7)	0.0020 (7)	0.0020 (7)
C10	0.0287 (11)	0.0383 (12)	0.0259 (11)	0.0009 (10)	−0.0053 (8)	0.0023 (9)
C11	0.0259 (9)	0.0184 (9)	0.0181 (9)	−0.0040 (7)	0.0012 (7)	0.0020 (7)
C2	0.0230 (9)	0.0191 (9)	0.0197 (9)	−0.0042 (7)	0.0031 (7)	0.0015 (7)
C3	0.0250 (9)	0.0242 (10)	0.0232 (10)	−0.0015 (8)	−0.0014 (8)	0.0037 (7)
C4	0.0259 (10)	0.0260 (10)	0.0305 (11)	0.0034 (8)	0.0048 (8)	0.0037 (8)
C5	0.0302 (11)	0.0218 (10)	0.0272 (10)	0.0009 (8)	0.0057 (8)	−0.0030 (8)
C6	0.0279 (10)	0.0231 (9)	0.0207 (10)	−0.0030 (8)	0.0024 (8)	−0.0034 (8)
C8	0.0233 (10)	0.0301 (11)	0.0171 (9)	−0.0007 (8)	−0.0021 (7)	−0.0034 (8)
C9	0.0247 (10)	0.0268 (10)	0.0210 (10)	0.0005 (8)	0.0003 (7)	−0.0008 (8)
N13	0.0271 (9)	0.0319 (9)	0.0220 (9)	0.0020 (7)	0.0004 (7)	−0.0081 (7)
O12	0.0314 (7)	0.0257 (7)	0.0207 (7)	−0.0001 (6)	−0.0044 (5)	−0.0015 (5)
O7	0.0219 (6)	0.0295 (7)	0.0177 (7)	0.0040 (5)	−0.0025 (5)	−0.0043 (5)

### Geometric parameters (Å, º)

**Table:**

C1—C2	1.407 (2)	C6—H6	0.927 (19)
C1—C6	1.393 (2)	C8—C9	1.505 (3)
C10—H10A	1.00 (2)	C8—H8A	0.987 (18)
C10—H10B	1.03 (2)	C8—H8B	1.01 (2)
C10—H10C	0.98 (2)	C9—C10	1.525 (3)
C2—C11	1.502 (2)	C9—H9A	0.99 (2)
C2—C3	1.398 (2)	N13—C11	1.334 (2)
C3—C4	1.385 (3)	N13—H13A	0.94 (2)
C3—H3	0.951 (18)	N13—H13B	0.93 (2)
C4—H4	0.996 (19)	O12—C11	1.244 (2)
C5—C4	1.386 (3)	O7—C1	1.370 (2)
C5—H5	0.94 (2)	O7—C8	1.444 (2)
C6—C5	1.383 (3)		
			
C1—C6—H6	119.9 (11)	C8—C9—H9A	109.2 (11)
C1—C2—C11	125.52 (15)	C8—C9—C10	110.78 (16)
C1—O7—C8	118.44 (13)	C9—C10—H10C	111.9 (14)
C10—C9—H9B	110.3 (11)	C9—C10—H10B	110.3 (12)
C10—C9—H9A	110.5 (11)	C9—C10—H10A	111.6 (12)
C11—N13—H13B	117.9 (12)	C9—C8—H8B	110.7 (11)
C11—N13—H13A	118.3 (12)	C9—C8—H8A	110.0 (11)
C2—C3—H3	117.9 (11)	H10A—C10—H10C	106.8 (18)
C3—C4—H4	121.4 (12)	H10A—C10—H10B	111.4 (17)
C3—C4—C5	118.80 (17)	H10B—C10—H10C	104.6 (18)
C3—C2—C11	116.41 (15)	H13A—N13—H13B	123.0 (17)
C3—C2—C1	118.04 (16)	H8A—C8—H8B	108.2 (15)
C4—C5—H5	120.5 (12)	H9A—C9—H9B	107.8 (15)
C4—C3—H3	120.2 (11)	N13—C11—C2	119.34 (15)
C4—C3—C2	121.97 (17)	O12—C11—C2	119.37 (15)
C5—C6—H6	120.1 (11)	O12—C11—N13	121.30 (17)
C5—C4—H4	119.7 (12)	O7—C8—H8B	110.2 (11)
C5—C6—C1	120.01 (17)	O7—C8—H8A	110.9 (11)
C6—C5—H5	118.5 (12)	O7—C8—C9	106.87 (14)
C6—C5—C4	121.00 (18)	O7—C1—C6	122.48 (16)
C6—C1—C2	120.17 (16)	O7—C1—C2	117.33 (15)
C8—C9—H9B	108.2 (10)		

### Hydrogen-bond geometry (Å, º)

**Table:**

*D*—H···*A*	*D*—H	H···*A*	*D*···*A*	*D*—H···*A*
N13—H13*A*···O7	0.94 (2)	1.95 (2)	2.669 (2)	132.2 (15)
N13—H13*B*···O12^i^	0.93 (2)	1.98 (2)	2.911 (2)	173.5 (19)
C6—H6···O12^ii^	0.927 (19)	2.517 (18)	3.442 (2)	175.8 (14)
C5—H5···C11^iii^	0.94 (2)	2.63 (2)	3.528 (2)	160.5 (17)

Symmetry codes: (i) −*x*, −*y*, −*z*+1; (ii) *x*+1/2, −*y*+1/2, *z*+1/2; (iii) −*x*−1/2, *y*+1/2, −*z*+3/2.
